# Management of Thrombosis Risk in a Carrier of Hemophilia A with Low Factor VIII Levels with Atrial Fibrillation: A Clinical Case and Literature Review

**DOI:** 10.1155/2018/2615838

**Published:** 2018-09-05

**Authors:** Nigel P. Murray, Lorena Muñoz, Simona Minzer, Marco Antonio Lopez

**Affiliations:** ^1^Consultant Hematologist, Dept of Medicine, Hospital de Carabineros, Simon Boliovar 2200, Nuñoa, 7770199 Santiago, Chile; ^2^Profesor Hematology, Faculty of Medicine, University Finis Terrae, Pedro de Valdivia 1509, Providencia, 7501015 Santiago de, Chile; ^3^Consultant Internal Medicine, Dept of Medicine, Hospital de Carabineros, Simon Boliovar 2200, Nuñoa, 7770199 Santiago de, Chile; ^4^Professor Medicine, Faculty of Medicine, University Mayor, Camino La Piramide 5750, Huechuraba, 8580745 Santiago de, Chile; ^5^Dept of Medicine, Hospital de Carabineros, Simon Boliovar 2200, Nuñoa, 7770199 Santiago de, Chile

## Abstract

Nonvalvular atrial fibrillation (AF) is a common age-related arrthymia and a leading cause of stroke in the elderly; with an aging hemophilia population, the number of patients developing AF is increasing. There are no controlled trials on thromboprophylaxis in this group of patients, only consensus opinion was based on small case reports. We present a female patient, carrier for hemophilia and with clinically moderately severe hemophilia who developed FA. We discuss the literature with respect to this group of patients and current recommendations for thromboprophylaxis.

## 1. Introduction

Nonvalvular atrial fibrillation (AF) is a common age-related arrthymia and a leading cause of stroke in the elderly [1]. In patients with AF, 80% of ischemic stroke events result in death or disability, and one-year mortality in these patients approaches 50% [2, 3]. To prevent thromboembolic complications of AF, life-time anticoagulation is recommended based on the projected risk of embolic events using the CHADS_2_ score [4] or the CHA_2_DS_2_-VASc score [5].

With advances in hemophilia care, the life expectancy of non-HIV hemophilia patients exceeds 70 years [6]. As the hemophilia population ages, the numbers of these patients presenting with age-related comorbidities such as AF is increasing. At present, there are no clinical trial data to support recommendations for anticoagulation in hemophilia patients with AF.

Hemophilia A is a rare X-linked hemorrhagic disorder that typically affects men; women are usually asymptomatic carriers but are reported to have a wide range of plasma concentrations of Factor VIII and as such may be carriers of hemophilia A with a low FVIII.

We report the case of a woman with hemophilia who at the age of 88 years developed AF, the management used and review of the literature.

## 2. Clinical Case

In 1978, a 41-year-old woman was diagnosed as a hemophilia carrier with a low FVIII level; from the age of 13, she had suffered menorrhagia of up to 8 days each month. In addition, she had suffered five episodes of hematemesis associated with epigastric pain and abundant hemorrhages during the births of her two sons and daughter and after dental extractions requiring red cell transfusions. The two sons had a history of “easy bleeding,” and the daughter did not have such characteristics.

At the age of 12 years, the two sons were diagnosed with hemophilia A; the results of the tests taken in 1978 are shown in Table 1. A chromosome analysis showed the normal 46XX karyotype. Both the patients' parents had died but there was no clinical history that the father had suffered from “bleeding problems”. No mutational analysis was carried out to confirm the diagnosis of hemophilia A.

From the time of the diagnosis, she had been treated with cryoprecipiate and later Factor VIII concentrates as necessary for dental extractions and a cholecystectomy. Her hemophilia carrier state with low FVIII level was defined as moderately severe.

In 2006, she was reassessed; the FVIII was 17%, Factor von Willebrand 104%, and Ristocetin CoFactor 110%. Her hemophilia carrier state with low FVIII level was redefined as mildly severe.

The patient presented in 2017 with increasing dyspnea, orthopnea, and tachycardia of 3-week duration. Chest X-ray was consistent with heart failure, and the ECG showed atrial fibrillation with a rapid ventricular response. She had a history of well-controlled hypertension and was taking Losartan 50 mg/day, was not diabetic, and did not have a history of stroke. 3D echocardiography showed a dilated left auricular, 22 mm^3^ with preserved ejection fraction and no valvular disease. She was treated with amiodarone and a beta-blocker; with an adequate ventricular response, amiodarone was suspended. Renal and liver function tests were normal.

Her CHADS_2_-VASC (congestive heart failure (C), hypertension (H), age ≥ 75 years (A), diabetes (D), stroke, transient ischemic attack or prior thromboembolic disease (S_2_) was 3 for age, sex, and hypertension arterial) [7], and a HAS-BLED score of 2 (hypertension (H), abnormal renal/liver function (A), stroke (S), bleeding history or predisposition (B), elderly (E), and drugs/alcohol (D)) [8]. Labile INR score was not included as the patient was being considered for anticoagulation. She was started on aspirin 100 mg/day as well as omeprazole 20 mg/day for the previous history of gastrointestinal bleeding. In this case, pulmonary vein isolation was not accepted by the patient due to its high cost.

At present, with a follow-up of 11 months, there has been no bleeding (stable hemoglobin) or gastric symptoms, and the AF rate is well controlled.

## 3. Discussion

At present, there are no guidelines or randomized trials to guide the management of patients with hemophilia and cardiovascular disease, and expert consensus based on case reports and observational studies are the only resource for treating physicians [9, 10]. With the use of recombinant FVIII, the life expectancy of hemophilia patients has increased, and as such the population at risk of developing atrial fibrillation has increased [10]. Considerations for the long-term reduction of stroke risk in these patients must be addressed. The prevalence of AF in hemophiliac patients increases with age, with a reported prevalence of 3.4% in those older than 60 years compared with 0.2% in younger patients [11].

The reduction of stroke risk in patients with atrial fibrillation involves the risk benefit of anticoagulation, the risk profile using CHADS_2_ or CHADS_2_-VASc scores, or HAS-BLED profile. In patients with hemophilia, the bleeding risk is dominant and algorithms have been proposed to consider whether anticoagulation is advisable [10]. Patients are stratified according to CHADS_2_-VASC risk and FVIII levels; a score of ≥3 being used as a cutoff point to recommend prophylactic anticoagulation. Although there are no data to validate a cutoff value in hemophiliac patients, a consensus agreement of ≥2 was suggested as patients are normally male and less than 75 years; in our case, the patient was female. It has also been recommended that the level of FVIII considered safe for considering anticoagulation should be lowered from 30% to 20% [12]. Bleeding has been reported in patients treated with warfarin and FVIII levels <205 [11–13], whereas in patients with FVIII levels >20%, the use of warfarin with hemorrhagic complications has been reported [14, 15]. However, this threshold should be evaluated on an individual basis.

With the new direct oral anticoagulants, there is a reported increased safety profile, especially with regard to intracerebral hemorrhage [16]. It has been suggested to the lower the dose of 10 mg daily of rivaroxaban oral Xa inhibitor that may be more appropriate and safer than the use of vitamin K antagonists [12]. It has been recommended that, in patients with a FVIII level of <20%, anticoagulation is contra-indicated. In these patients, the use of low dose aspirin has not been reported to increase the risk of hemorrhages [12, 14]. However, the 2012 European guidelines omitted the use of aspirin in the management of FA [17].

It has also been suggested that low-dose aspirin should be used in patients with FVIII levels of 5–30%, in those with levels of 1–5% but with a CHADS_2_ score of ≥2 and those severe hemophiliacs receiving FVIII prophylaxis [18].

Alternates include catheter ablation (pulmonary vein isolation); patients are managed with replacement therapy and anticoagulation for three months postablation, and this has been reported to be comparable to long-term anticoagulation in the prevention of thromboembolic events [19]. Percutaneous closure of the left atrial appendage is another approach in patients with a high bleeding risk [20]. However, in many countries, these approaches are not routinely available. In our case, prophylaxis with low-dose aspirin was commenced.

The majority of severe or moderate phenotype women with hemophilia are heterozygous carriers of one mutant hemophilia allele with a normal female karyotype (46XX), which correlates with a skewed X chromosome inactivation pattern [21]. However, the degree of skewing does not fully predict plasma FVIII levels [22]. The pattern of skewing varies among tissue types and may vary with age, a phenomenon potentially accentuated by aging [23]. This may be one explanation of the different FVIII levels detected in this clinical case or differing methods manual versus automatic accounts for the different Factor VIII levels.

Mutation analysis to confirm the diagnosis of hemophilia A was not carried out; in view that one of the sons had a significantly higher FVIII level (19%), the possibility of von Willebrand disease type 2N (Normandy) arises. In type 2N von Willebrand disease, a normal von Willebrand factor activity does not rule out the diagnosis. However, mutation analysis is not available in Chile.

## 4. Conclusions

This case highlights that, firstly, each hemophilic patient who develops atrial fibrillation must be evaluated on an individual basis, taking into account the risk factors and benefits of anticoagulation. The levels of FVIII in female carriers may vary with time and thus change the risk/benefit assessment. This is important in carriers of hemophilia A with low FVIII levels, since an increase in FVIII levels may decrease the risk of bleeding.

Although aspirin is recommended for some patients, there are no studies to confirm its efficacy in these patients. Consensus guidelines are the best recommendation at the present time.

## Figures and Tables

**Table 1:** Coagulation tests of the patient and family.

	Normal range	Patient	Son A	Son B	Daughter
Coagulation time	<15 minutes	12	13	12	13
Bleeding time	<7 minutes	5	4	2.5	2
Platelet count	150–300	205	190	210	239
Prothrombin time (s)	12–15 seconds	12.5	11	14	12
FVIII fraction procoagulant fraction antigen	50–150% Present	4 Present	7 Present	19 Present	110 Present
Platelet aggregation with ristocetin (%)	>70	80	—	—	—
Platelet adhesion (%)	>20	67	—	—	—
